# Attitude towards death in family members of adolescents who applied to the crisis care room of a child psychiatric clinic due to suicidal thoughts

**DOI:** 10.1192/j.eurpsy.2022.854

**Published:** 2022-09-01

**Authors:** M. Bebchuk, D. Dovbysh, S. Timoshenko, M. Talmach, Y. Zhorina, A. Diachenko, R. Rabadanova

**Affiliations:** 1 Scientific-Practical Сhildren’s and Adolescents Mental Health Center n.a. G. Sukhareva, Moscow Department of Health Care, Clinical Psychology, Moscow, Russian Federation; 2 Federal State Autonomous Educational Institution of Higher Education I.M. Sechenov First Moscow State Medical University under the Ministry of Health of the Russian Federation (Sechenov University), Pedagogy And Clinical Psychology, Moscow, Russian Federation

**Keywords:** Attitude towards death, Crisis care, suicidal thoughts

## Abstract

**Introduction:**

The study of ideas about death and the mechanisms of their formation in adolescents with suicidal behavior is an important task for both diagnosis and psychotherapy. The parents’ notions of death, and the prohibition on discussing the topic of death in the family, can significantly distort the adolescent’s relationship with death.

**Objectives:**

Investigate differences in attitudes towards death in parents and adolescents who apply to the crisis care room of a child psychiatric clinic.

**Methods:**

The study involved 90 adolescents with their parents who applied to the crisis outpatient care system. The following questionnaires were offered: Death Attitude Profile-Revised, Death Anxiety Scale, Experiences in Close Relationships-Revised, Multidimensional Scale of Perceived Social Support, GAD-7.

**Results:**

Significant differences were found for most of the scales in the samples of adults and adolescents, of particular interest to us were the scales “death avoidance” (W=317,500,z=3,089,p=0,002) and “perception of family support» (W=288,500,z=3,393,p=0,001). For the group of adolescents, there are higher indicators on the scales of anxiety (W=61,000,z=-2,546,p=0,011), anxiety in close relationships (W=83,000,z=-2,549,p=0,011), avoidance of close relationships (W=28,000,z=-3,870,p=0,000), and death as a means of escape (W=54,500,z=-3,076,p=0,002).

**Conclusions:**

In families of adolescents with suicidal behavior, the severity of dysfunctional patterns of building close relationships and the presence of sharply opposite positions in relation to death in children and parents are observed without the possibility of discussing this topic.

**Disclosure:**

No significant relationships.

